# Scleral Thickness in Eyes with Pachychoroid Pigment Epitheliopathy Accompanied by Keratoconus

**DOI:** 10.1016/j.xops.2025.100808

**Published:** 2025-04-26

**Authors:** Paolo Forte, Alessandro Feo, Hideki Koizumi, Enrico Borrelli, Riccardo Manocchio, Luca Di Cello, Francesco Biagini, Francesco Macocco, Gabriele Drago, Giovanni Forte, Aldo Vagge, Christian Gianoglio, Vincenzo Fontana, Michele Iester, Massimo Nicolò, Mario R. Romano, Chiara Bonzano

**Affiliations:** 1 Eye Unit, IRCCS Ospedale Policlinico San Martino, Genoa, Italy; 2 DINOGMI, University of Genoa, Genoa, Italy; 3 Department of Biomedical Sciences, Humanitas University, Milan, Italy; 4 Department of Ophthalmology, Graduate School of Medicine, University of the Ryukyus, Okinawa, Japan; 5 Department of Surgical Sciences, University of Turin, Turin, Italy; 6 Department of Ophthalmology, “City of Health and Science” Hospital, Turin, Italy; 7 Department of Clinical-Surgical, Diagnostic and Pediatric Sciences, University of Pavia, Italy; 8 Department of Naval, Electrical, Electronics and Telecommunications Engineering (DITEN), University of Genoa, Genoa, Italy; 9 Department of Mathematics, University of Genoa, Genoa, Italy; 10 Department of Ophthalmology, Eye Unit Humanitas Gavazzeni-Castelli, Bergamo, Italy

**Keywords:** Pachychoroid pigment epitheliopathy, Keratoconus, Sclera, Pachychoroid, OCT

## Abstract

**Purpose:**

To assess the prevalence of pachychoroid pigment epitheliopathy (PPE) in eyes with keratoconus (KC) and investigate its correlation with corneal, choroidal, and scleral indices with multimodal imaging.

**Design:**

An exploratory, cross-sectional, cohort study.

**Subjects:**

One hundred consecutive patients affected with KC.

**Main Outcome Measures:**

Scleral stromal thickness, PPE prevalence, and their associations with corneal and choroidal parameters.

**Methods:**

Demographic data, corneal collagen cross-linking, anamnestic records, and clinical findings were collected. Imaging protocol included OCT (Spectralis HRA+OCT; Heidelberg Engineering), corneal topography (TMS-4N, Tomey), corneal pachymetry (RTVue-XR Avanti, Optovue), and axial length (AXL) measurement (OA-2000, Tomey). Anterior scleral stromal thickness was measured in the horizontal gaze positions 6 mm posteriorly to the scleral spur (Spectralis HRA+OCT; linear 20° scan, 1024 A-scan per second). Odds ratios (ORs) and corresponding 95% confidence limits (95% CLs) were estimated through logistic regression analysis to evaluate the association between each study parameter and PPE. To accommodate for the potential clustering effect due to within-patient correlated eye data, a generalized estimating equation procedure was applied to regression analysis. Additionally, a decision tree machine learning model with K-fold cross-validation was employed to predict PPE.

**Results:**

Eighty-five Caucasian patients were eligible for analysis (mean age: 34.2 years, standard deviation: 8.7). The prevalence of PPE was 10.5% (95% CL: 4.9/19.1%; 9/85 patients; 11/170 eyes; 2 bilateral cases). Significant predictors for PPE according to logistic regression were choroidal thickness (OR: 4.51; 95% CL: 1.50/13.6 for 50 μm increments; *P* = 0.007), age (OR: 4.61; 95% CL: 1.30/16.4 for 10-year increments; *P* = 0.018), and scleral stromal thickness (OR: 7.48: 95% CL: 1.69/33.1 for 25 μm increments; *P* = 0.008). Sex, AXL, corneal curvature, and astigmatism parameters did not show significant discriminant ability (*P* > 0.05). Collagen cross-linking treatment was performed in a comparable proportion between the 2 groups (73.6% vs. 63.4% in PPE and non-PPE, respectively).

**Conclusions:**

Our study identifies increased scleral thickness as the key predictor of PPE in KC patients, followed by choroidal thickening and increased age. These findings provide new insights into the role of scleral biomechanics in KC eyes with PPE.

**Financial Disclosure(s):**

The author(s) have no proprietary or commercial interest in any materials discussed in this article.

Choroidal thickening, a hallmark of the pachychoroid disease spectrum (PDS), is increasingly recognized in eyes with keratoconus (KC).^1^ Keratoconus is a bilateral, typically asymmetric, ectatic disease characterized by progressive corneal thinning and protrusion. While the precise etiology of KC remains uncertain, multiple factors contribute to its pathogenesis, including genetic predisposition, ultraviolet light exposure, mechanical stress from compulsive eye rubbing, and alterations in collagen and extracellular matrix composition. These pathogenic mechanisms may extend beyond the anterior segment, potentially affecting the anatomical and functional characteristics of the posterior segment.^2^

Since initial reports by Eandi et al in 2008,^3^ KC has been identified as an anatomical risk factor for central serous chorioretinopathy.^4^^,^^5^ More recently, Feo et al^6^ described an association between KC and pachychoroid pigment epitheliopathy (PPE), a component disease of the PDS.^7^ Their investigation of corneal topometric indices, retinal pigment epithelium (RPE) abnormalities, and subfoveal choroidal thickness (SFCT) suggested that a combination of shared corneal and chorioretinal collagen dysfunction affecting both the RPE and choroidal vessels, together with subtle inflammatory and immune-mediated processes, might represent the pathogenic link between these 2 disorders.^6^ The phenotypic manifestations of PPE comprise various RPE abnormalities, primarily characterized by RPE thickening and small pigment epithelial detachments, occurring in the absence of subretinal fluid.^8^

A potentially underappreciated factor playing a role in PDS is the thickening of the scleral stromal layer, a risk factor initially reported by the Koizumi group in Japanese cohorts.^9^ We recently validated the correlation between increased scleral stromal thickness and central serous chorioretinopathy in Caucasian patients.^10^ However, additional evidence is needed to establish this risk factor in the pathophysiology of PPE and PDS.

The present study aimed to explore the in vivo anatomical features potentially contributing to the PPE occurrence in keratoconic eyes, with particular emphasis on scleral changes. Additionally, we implemented a supervised machine learning model to identify significant features associated with PPE development in these patients.

## Methods

### Study Design and Population

This exploratory, cross-sectional cohort study was conducted at the University of Genoa (Italy). This study adhered to the tenets of the Declaration of Helsinki. The regional ethics committee of the Liguria region (CER) was notified about this retrospective study, and informed consent was obtained from all patients.

Consecutive patients with a history of epi-off corneal collagen cross-linking treatment (CXL) previously performed by a single surgeon (C.B.) were recruited from the University Eye Clinic at San Martino IRCCS Hospital, Genoa, Italy.

The study included 100 consecutive Caucasian patients diagnosed with KC, who were screened for the presence of PPE. Exclusion criteria included (1) history of any other chorioretinal or optic nerve disorders; (2) relevant optical media opacities or insufficient fixation to allow high-quality imaging; (3) any history or active signs of scleritis or episcleritis; (4) inability to measure scleral stromal thickness due to suboptimal image quality; (5) current pregnancy or history of pregnancy in the previous 12 months; (6) history of any corneal transplantation or other ocular surgical procedures, except for CXL, if performed at least 6 months before image acquisition, in order to prevent potential choroidal changes associated with the procedure^11^; (7) exposure to previous or current steroid treatment; (8) poor quality of OCT or corneal topographic images. Because subjects with a prior history of central serous chorioretinopathy might display signs similar to PPE after subretinal fluid resolution, patients with evidence or a history of prior subretinal fluid accumulation were also excluded.

### Data Collection

Demographic data, time between CXL treatment and study visit in years when applicable, and anamnestic records (history of atopy and exposure to corticosteroid drugs) were collected by an independent ophthalmologist (L.D.C.).

All enrolled patients underwent a multimodal imaging evaluation. F.M. and G.D. performed best-corrected visual acuity measurements using logMAR charts, corneal topography (TMS-4N, Tomey), and corneal pachymetry (RTVue-XR Avanti, Optovue Inc). R.M. and F.B. performed axial length (AXL) measurements (OA-2000, Tomey), pseudocolor fundus photography (MultiColor), and structural OCT (Spectralis HRA+OCT; Heidelberg Engineering). Indocyanine green angiography (Spectralis HRA+OCT; Heidelberg Engineering) was performed in patients showing multiple loci of PPE. Scleral stromal thickness measurements were acquired in the 2 horizontal gaze positions (temporal and nasal) using a scanning laser ophthalmoscope (Spectralis HRA+OCT; Heidelberg Engineering). The imaging protocol included a linear 20° scan (1024 A-scans per second), with enhanced contrast before image analysis.

### OCT Grading

OCT images were first reviewed for eligibility by 2 independent graders (P.F. and A.F.). These graders, who were masked to visual results and corneal metrics, independently evaluated each eligible eye for qualitative and quantitative features. The median and interquartile range values were employed for statistical analysis for quantitative measurements. The presence of PPE was binarily defined as absent or present by the detection of RPE abnormalities on OCT imaging confirmed by characteristic findings on fundus autofluorescence and large choroidal vessels in the area immediately underneath RPE abnormalities. Areas of abnormal fundus autofluorescence were further analyzed with OCT to identify RPE alterations, including RPE thickening, hyperreflective RPE spikes, and pigment epithelial detachment, in the absence of pachychoroid-related exudation, such as subretinal fluid or subretinal hyperreflective material.^12^^,^^13^ For all quantitative measurements, including scleral thickness, the mean value from both graders was used for statistical analysis. For categorical features, when readers disagreed, an additional assessment was performed by a third senior author (M.N.).

**Figure 1:**
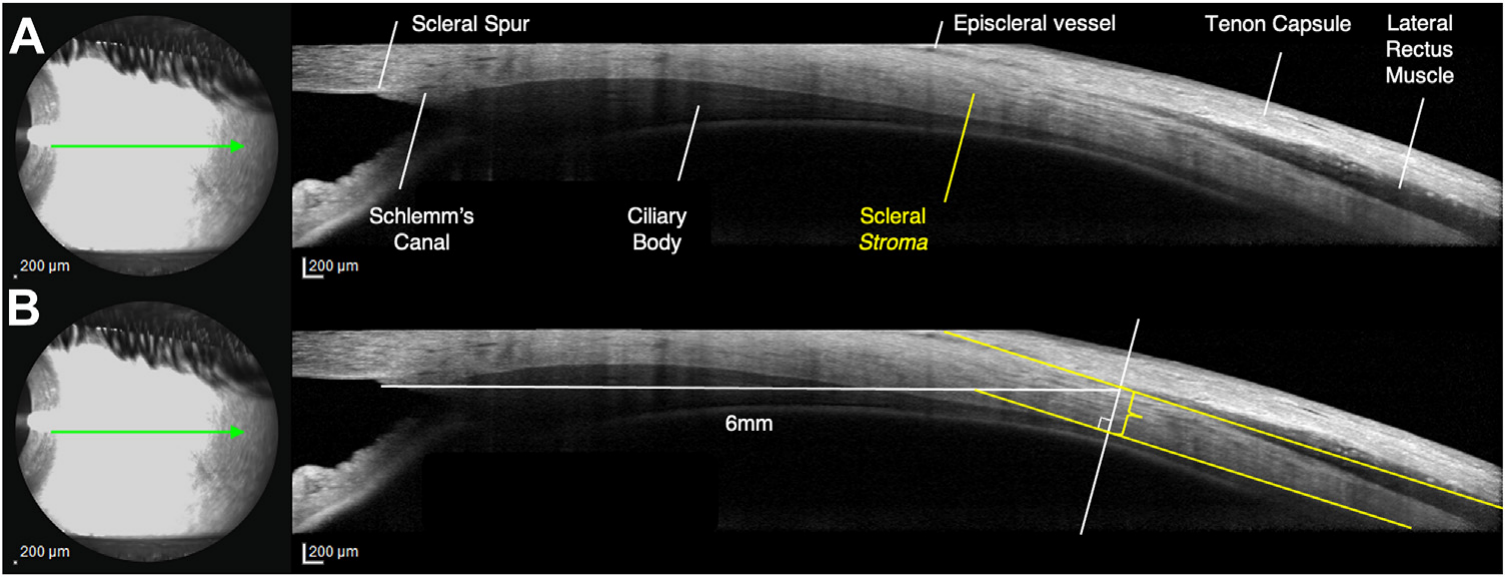
Adapted with permission from Forte et al, 2024^10^—Anterior scleral thickness measurement was obtained in the horizontal gaze positions (temporal and nasal). **A,** Key anatomical landmarks included the SS, episcleral vessels, and the recti muscles visualized as hyporeflective bands. **B,** Scleral *substantia propria* thickness, also termed the *stromal* thickness (yellow *brace*), was specifically assessed at a point 6 mm posterior to the SS. Measurement consisted of manually measuring the vertical distance between the chorioscleral interface and the corresponding hyporeflective recti muscles' bodies or perimysia (*yellow line* drawn perpendicularly to the inner wall of the scleral stroma), as described by Imanaga et al. SS = scleral spur.

Subfoveal choroidal thickness was measured manually using the OCT caliper tool as the vertical distance between the hyperreflective RPE-Bruch membrane complex and the hyperreflective sclero-choroidal junction. Subfoveal choroidal thickness measurement was not directly used as a diagnostic component for PPE but rather as an anatomical parameter for comparison between groups and for statistical analysis. Scleral stromal thickness measurements followed methods described in our previous study (Forte et al, 2024),^10^ at 6-mm eccentricity from the scleral spur^12^: after identifying the scleral spur and the 2 horizontal *recti* muscles as hyporeflective bands,^14^ anterior scleral stromal thickness was measured as the vertical distance between the sclero-choroidal interface and the corresponding recti muscles body or perimysia at nasal and temporal locations in horizontal gaze, as displayed in Figure 1. The reliability of this measurement approach has been validated in previous studies, with reported intraclass correlation coefficients exceeding 0.90 for both intraobserver and interobserver assessments.^15^^,^^16^

### Statistical Analysis

Metric parameters were described using the median and interquartile range. For group comparisons, the nonparametric Mood median test was used to compare the non-PPE and PPE groups. Odds ratios (ORs) and corresponding 95% confidence limits (95% CLs) were estimated through logistic regression analysis to evaluate the association between each study item and PPE. To accommodate for the potential clustering effect due to within-patient correlated eye data, generalized estimating equations procedure was applied to regression analysis. Covariates considered for the logistic regression modeling were selected according to a critical review of previous literature (P.F.) regarding scleral structure and KC-specific features. Specifically, the biomechanical and stiffness properties of the sclera are associated with AXL^17^ and age.^18^ To further investigate the contribution of ocular parameters to scleral thickness, a linear regression modeling of mean scleral thickness was obtained in the non-PPE group with the following explanatory variables: AXL, steep keratometry, SFCT, sex, and age. All statistics derived from regression modeling were accompanied by the corresponding 95% CLs, and a 2-tailed *P* < 0.05 was considered statistically significant. All data analyses were conducted using Stata Statistical Software: Release V.17 (StataCorp. 2021).

#### Machine Learning Modeling

We implemented a supervised decision tree (DT) machine learning model to predict the presence of PPE in patients. This model operates on a tree-like structure, where each node represents a decision based on a specific attribute. Branches represent the outcomes of these tests, and leaf nodes convey the final classification. The algorithm recursively divides the dataset into subsets, utilizing features that best differentiate between classes.^19^ The input features for the DT are the same as those used in the statistical model.

To ensure robust model evaluation and avoid overfitting, we employed a stratified leave-one-out cross-validation technique.^20^ In this iterative process, the dataset was divided into 11 subsets, each time excluding 1 of the 11 positive samples from the training set. For each fold, the model was trained and evaluated 11 times, with each iteration using a different subset as the test set and the remaining subsets as the training set. This approach ensured that each test set contained 1 negative sample and led to the creation of 11 unique DTs, each representing a distinct testing scenario. In the feature analysis, each DT provided feature importance scores, quantifying the contribution of each feature to the model's decision-making process. These scores were collected for each run of the cross-validation. To address eventual class imbalance, we combined stratified leave-one-out cross-validation with the DT's built-in class weighting mechanism, thereby minimizing bias and ensuring appropriate representation of both classes. To obtain a comprehensive view of feature relevance, the average importance score across the 11 folds was calculated for each feature. This average provides a representative measure, highlighting the significance of each feature in the context of PPE prediction.

## Results

### Descriptive Analysis

A total of 85 patients were eligible for analysis (mean age: 34.2 years; standard deviation: 8.7 years; range: 19–58 years). Reasons for exclusion were history of penetrating keratoplasty (n = 2), prior corticosteroid exposure (n = 6), and poor imaging quality or missing examinations in the screening protocol (n = 7). Medical history was positive for eye rubbing in 42.3% and atopy in 22.3% of patients.

The prevalence of PPE was 10.5% (95% CL: 4.9/19.1%; 9/85 patients; 11/170 eyes; 2 bilateral cases). All PPE cases were asymptomatic and represented incidental findings without evidence of more advanced pachychoroid spectrum manifestations; no cases of polypoidal choroidal vasculopathy or pachychoroid neovasculopathy were identified in our screening process. For descriptive purposes only, we classified the 11 PPE-positive eyes using the Amsler–Krumeich system: 6/11 (54.5%) stage 1, 3/11 (27.3%) stage 2, 1/11 (9.1%) stage 3, and 1/11 (9.1%) stage 4; this distribution suggests that PPE can occur at any stage of KC severity. Table 1 provides an overview of age and gender distribution in PPE and non-PPE groups. Collagen cross-linking treatment showed a comparable proportion between the 2 groups (73.6% vs. 63.4% in PPE and no-PPE, respectively; *P* = 0.311), as summarized in Table 2.

**Table 1:** Demographic Characteristics of Non-PPE (Unaffected) and PPE Participants in the Study Cohort

Variable	Non-PPE		PPE		*P* Value
	*N*	%	*N*	%	
Age (yrs)					0.055
19–30	40	48.2	0	0.0	
31–40	29	34.9	4	44.4	
41–58	14	16.9	5	55.6	
Sex					0.165
Male	55	66.3	8	88.9	
Female	28	33.7	1	11.1	
Total patients	83	100.0	9	100.0	

PPE = pachychoroid pigment epitheliopathy.

**Table 2:** Distribution of CXL Treatment in Non-PPE (Unaffected) and PPE Participants

Variable	Non-PPE		PPE		*P* Value
	*N*	%	*N*	%	
CXL (yrs)					0.311
Never	42	26.4	4	36.4	
≥6	51	32.1	5	45.5	
<6	66	41.5	2	18.2	
Total eyes0	159	100.0	11	100.0	

CXL = collagen cross-linking.

**Figure 2:**
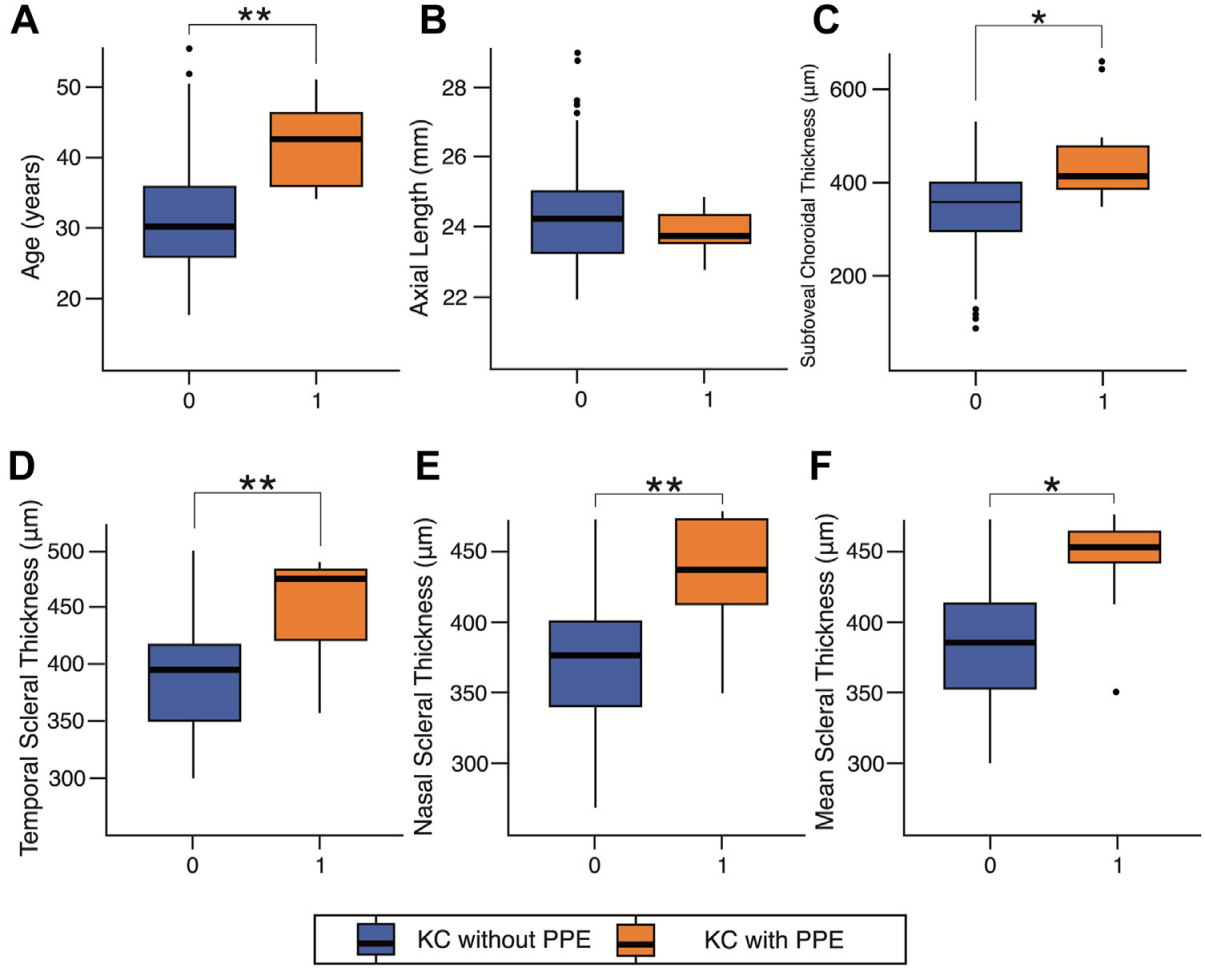
Box plots of clinical and anatomical parameters between KC with PPE (*orange*) and KC without PPE (*blue*) groups. Significant differences were observed in subfoveal choroidal thickness (*P* = 0.013), temporal scleral thickness (*P* = 0.010), nasal scleral thickness (*P* = 0.010), mean scleral thickness (*P* = 0.013), and age (*P* = 0.005). Importantly, no significant difference was found in axial length (*P* = 0.560). KC = keratoconus; PPE = pachychoroid pigment epitheliopathy.

A comparison of the clinical and anatomical characteristics between the 2 groups was shown in Table 3. Patients with PPE demonstrated significantly higher SFCT (417.0 vs. 349.0 μm; *P* = 0.013), temporal (474.0 vs. 394.0 μm; *P* = 0.010), nasal (439 vs. 374 μm; *P* = 0.010), mean scleral thickness (*P* = 0.013), and older age (40.5 vs. 30.6 years; *P* = 0.005), as shown in Figure 2. No significant differences were found in best-corrected visual acuity, central corneal thickness, thinnest corneal location, AXL, central mean retinal thickness, steep keratometry, flat keratometry, surface asymmetry index, and cylinder diopters.

**Table 3:** Comparison of Ocular Characteristics between Non-PPE and PPE Groups

Variable	Non-PPE			PPE			*P* Value
	*N*	Median	IQR	*N*	Median	IQR	
BCVA (logMAR)	159	0.0	0.1	11	0.0	0.2	0.825
CCT (μm)	159	496.0	57.0	11	486.0	69.0	1.000
TCL (μm)	159	466.0	59.0	11	461.0	77.0	1.000
Axial length (mm)	159	24.2	1.6	11	23.7	0.9	0.560
CMT (μm)	159	273.0	28.0	11	284.0	23.0	0.198
SFCT (μm)	159	349.0	101.0	11	417.0	102.0	0.013∗
Sclera temporal (μm)	159	394.0	67.0	11	474.0	64.0	0.010∗
Sclera nasal (μm)	159	374.0	62.0	11	439.0	63.0	0.010∗
Sclera mean (μm)	159	382.5	55.0	11	453.0	32.5	0.013∗
Ks (D)	159	47.5	5.9	11	48.7	8.50	0.533
Kf (D)	159	44.2	3.9	11	45.0	3.50	0.533
Sai (D)	159	1.75	1.56	11	1.50	0.80	1.000
Cyl (D)	159	3.52	3.36	11	3.90	3.70	1.000
Age (yrs)	83	30.6	10.6	9	40.5	9.72	0.005∗

BCVA = best-corrected visual acuity; CCT = central corneal thickness; CMT = central macular thickness; Cyl = cylinder; D = diopters; IQR = interquartile range; Kf = flat keratometry; Ks = steep keratometry; PPE = pachychoroid pigment epitheliopathy; Sai = surface asymmetry index; SFCT = subfoveal choroidal thickness; TCL = thinnest corneal location.

Values are presented as median and IQR.

*P* values were calculated using Mood median test.

∗ Statistically significant (*P* < 0.05).

Logistic regression analysis identified 3 significant predictors for PPE: choroidal thickness (OR: 4.51; 95% CL: 1.50/13.6 for 50 μm increments; *P* = 0.007), age (OR: 4.61; 95% CL: 1.30/16.4 for 10-year increments; *P* = 0.018), and scleral stromal thickness (OR = 7.48; 95% CL = 1.69/33.1 for 25 μm increments; *P* = 0.008). Sex, AXL, corneal curvature, and astigmatism parameters did not show significant discriminant ability (*P* > 0.05), as shown in Table 4.

**Table 4:** Logistic Modeling of PPE

Logistic Modeling of PPE			
Variable	OR	95% CL	*P* Value
Sex (female vs. male)	0.02	0.01/1.80	0.089
Axial length (Trend x 1 mm)	1.08	0.74/1.56	0.694
Ks (Trend × 3 D)	1.57	0.77/3.20	0.217
SFCT (Trend × 50 μm)	4.51	1.50/13.6	0.007∗
Age (Trend × 10 yrs)	4.61	1.30/16.4	0.018∗
Mean sclera thickness (Trend × 25 μm)	7.48	1.69/33.1	0.008∗

CL = confidence limit; Ks = steep keratometry; OR = odds ratio; PPE = pachychoroid pigment epitheliopathy; SFCT = subfoveal choroidal thickness.

∗ Statistically significant.

Among non-PPE patients, Table 5 reveals axial elongation significantly correlated with reduced mean scleral thickness (Δ: −2.60, 95% CL: −4.84/−0.36; *P* = 0.023). Corneal curvature (*P* = 0.885), gender (*P* = 0.717), and age-related trends (*P* = 0.311) were not significant predictors.

**Table 5:** Linear Regression Modeling of Mean Scleral Stromal Thickness in Unaffected (Non-PPE) Patients

Normal Modeling of Mean Scleral Thickness in PPE = 0			
Variable	Δ	95% CL	*P* value
Axial length (Trend × 1 mm)	−2.60	−4.84/v0.36	0.023∗
Ks (Trend × 3 D)	−0.27	−3.75/3.23	0.885
Gender (F vs. M)	2.91	−12.8/18.7	0.717
Age (Trend × 10 yrs)	4.62	−4.31/13.6	0.311
Constant	431.2	345.8/516.6	-

CL = confidence limit; Ks = steep keratometry; PPE = pachychoroid pigment epitheliopathy.

∗ Statistically significant.

**Figure 6:**
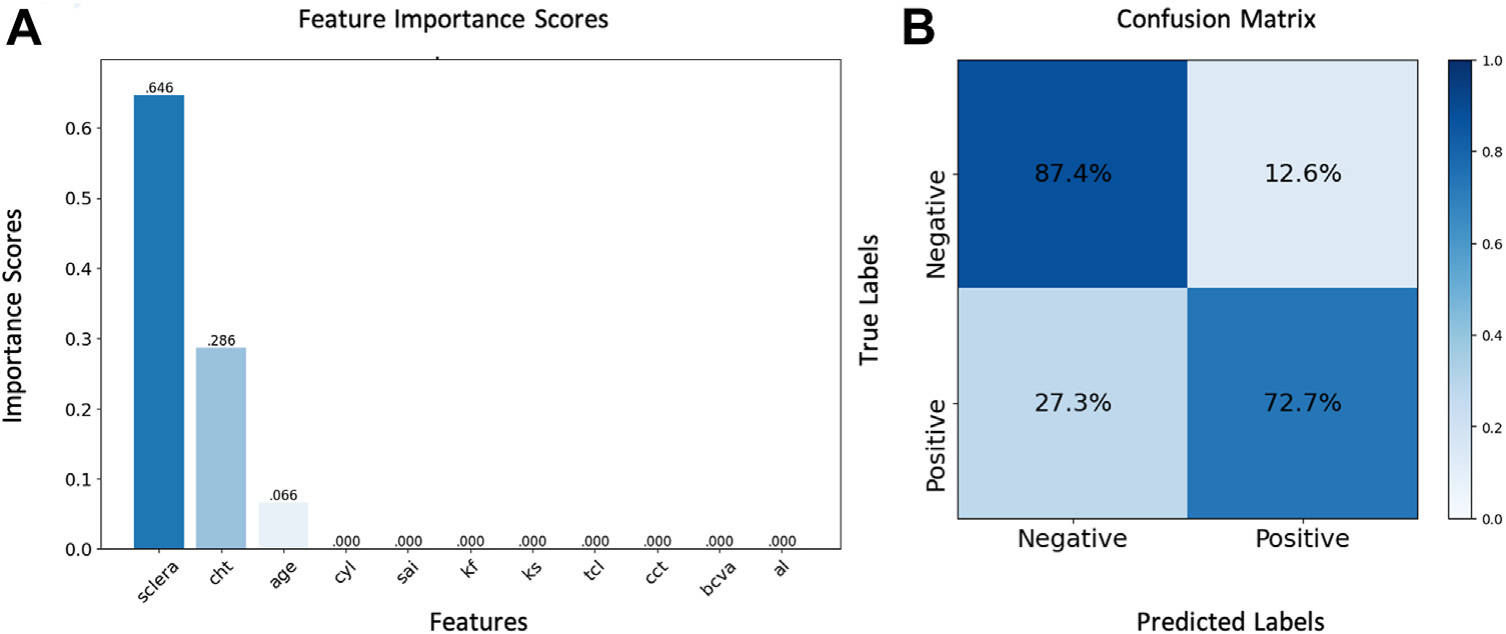
**A,** Feature importance score derived from the DT-supervised machine learning model and **(B)** confusion matrix depicting model performance in PPE prediction in KC patients. The algorithm using stratified leave-one-out cross-validation identified scleral thickness (0.646), choroidal thickness (0.286), and age (0.066) as the primary discriminative features. Model evaluation metrics showed high specificity (87.4%) but lower sensitivity (72.7%), reflecting the class imbalance in the dataset PPE prevalence. DT = decision tree; KC = keratoconus; PPE = pachychoroid pigment epitheliopathy.

The binary classification DT model further validated the results derived from logistic regression modeling. Specifically, among all 11 cross-validation models, 10 predicted PPE using only scleral and choroidal thickness information as features. Feature importance analysis identified scleral thickness as the dominant predictor, with a normalized Gini importance score of 0.646, followed by choroidal thickness (0.286) and age (0.066). In terms of model performance metrics, the models successfully identified 8 out of 11 cases of PPE but showed a false-negative rate of 27.3% (type II error) and a false-positive rate of 12.6% (type I error). The confusion matrix (Fig 6) indicated high specificity (87.4% true negatives) but lower sensitivity (72.7% true positives) in PPE detection, suggesting potential for model optimization, given the class imbalance in our series (PPE: 11/170 eyes). Both statistical approaches (logistic regression and machine learning) independently confirmed the predictive value of increased scleral stromal and choroidal thickness in PPE development.

## Discussion

Pachychoroid pigment epitheliopathy is considered the initial or precursor lesion of PDS disorders.^21^ The presence of PPE in keratoconic eyes was recently described by Feo et al,^6^ who identified typical PPE lesions in 17.9% (10 of 56 eyes) of 35 KC patients. In their study, SFCT was significantly higher in KC eyes compared to healthy eyes, but it did not differ across different KC stages or between KC eyes with and without PPE. Keratoconus was the only significant predictive factor for PPE in these eyes.^6^

**Figure 3:**
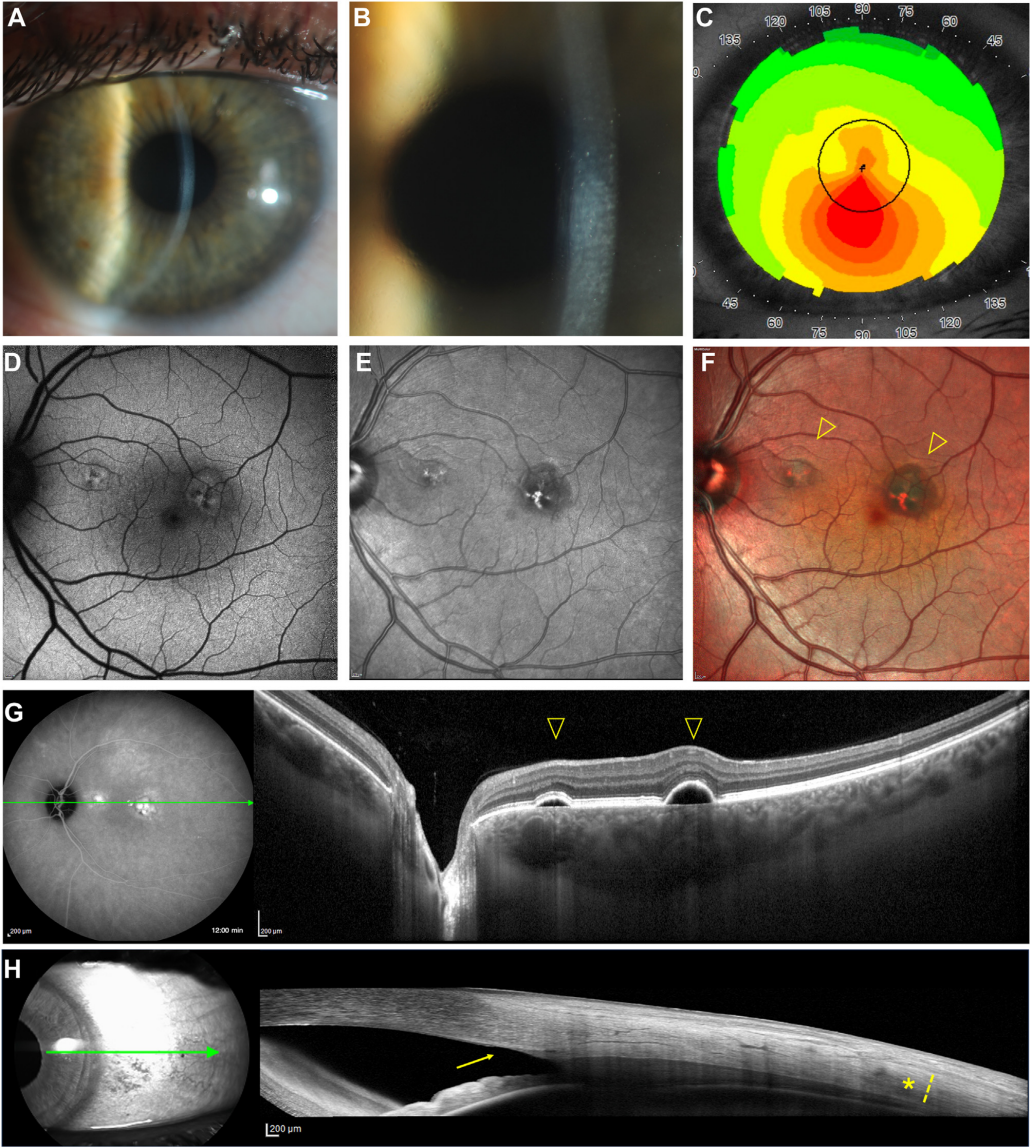
Multimodal imaging evaluation including scleral thickness in a male patient (43 years old) affected by KC with PPE. **A,** Slit-lamp photography and **(B)** high-magnification view showing corneal posterior stromal striae (*Vogt striae*). **C,** Corneal topography confirming the diagnosis of KC. **D,** Fundus autofluorescence, **(E)** infrared reflectance, **(F)** multicolor imaging, and **(G)** mid-frames (10:00 minutes) ICGA with corresponding OCT showing multifocal PEDs (*arrowhead*) overlying dilated choroidal vessels and posterior fluid accumulation in the outer choroid. **H,** In nasal gaze, the temporal scleral layers were visualized using anterior segment OCT, revealing scleral stromal thickness (*asterisk and dashed line*; 487 μm), 6 mm posterior to the scleral spur (*arrow*). Study eye axial length: 22.82 mm. ICGA = indocyanine green angiography; KC = keratoconus; PED = pigment epithelial detachment; PPE = pachychoroid pigment epitheliopathy.

**Figure 4:**
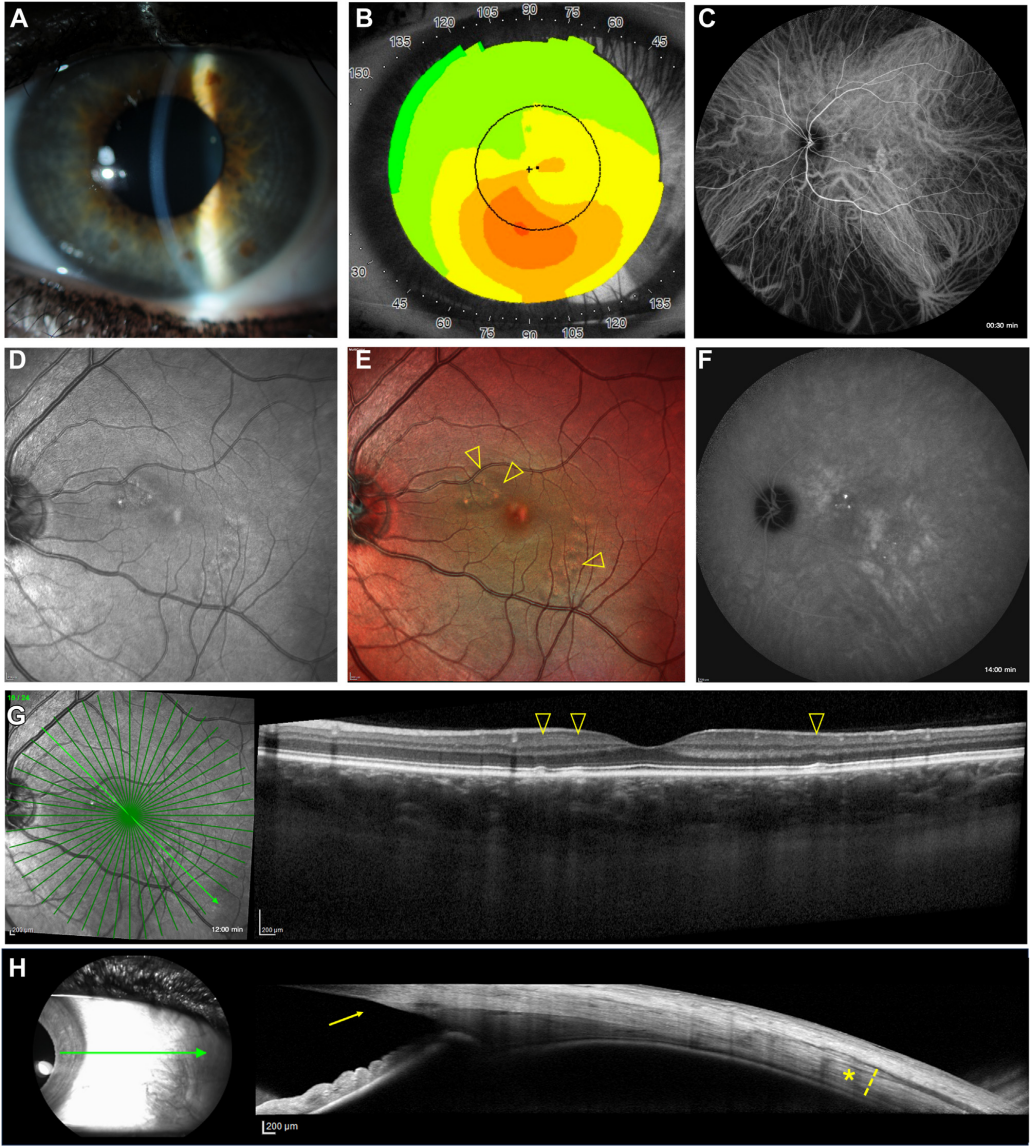
Multimodal imaging evaluation including scleral thickness in a female patient (35 years old) affected by KC with PPE. **A,** Slit-lamp photography and **(B)** corneal topography confirming the diagnosis of KC. **C,** Early frames (01:00 minutes) of ICGA showing choroidal vasculature. The horizontal watershed at the macula appears less pronounced, and the PPE lesions are predominantly located in the area drained by the inferotemporal vortex vein, which shows a mild degree of dilation. **D,** Infrared reflectance, **(E)** multicolor imaging, and **(F)** late-frames (20:00 minutes) of ICGA demonstrating multifocal PPE loci with choroidal hyperpermeability. **G,** Infrared reflectance with corresponding OCT revealing multifocal RPE elevations (*arrowhead*) and increased choroidal thickness. H, In nasal gaze, the temporal scleral layers were visualized using anterior segment OCT, revealing scleral stromal thickness (*asterisk and dashed line*; 480 μm), 6 mm posterior to the scleral spur (*arrow*). Study eye axial length: 23.11 mm. ICGA = indocyanine green angiography; KC = keratoconus; PPE = pachychoroid pigment epitheliopathy; RPE =retinal pigment epithelium.

**Figure 5:**
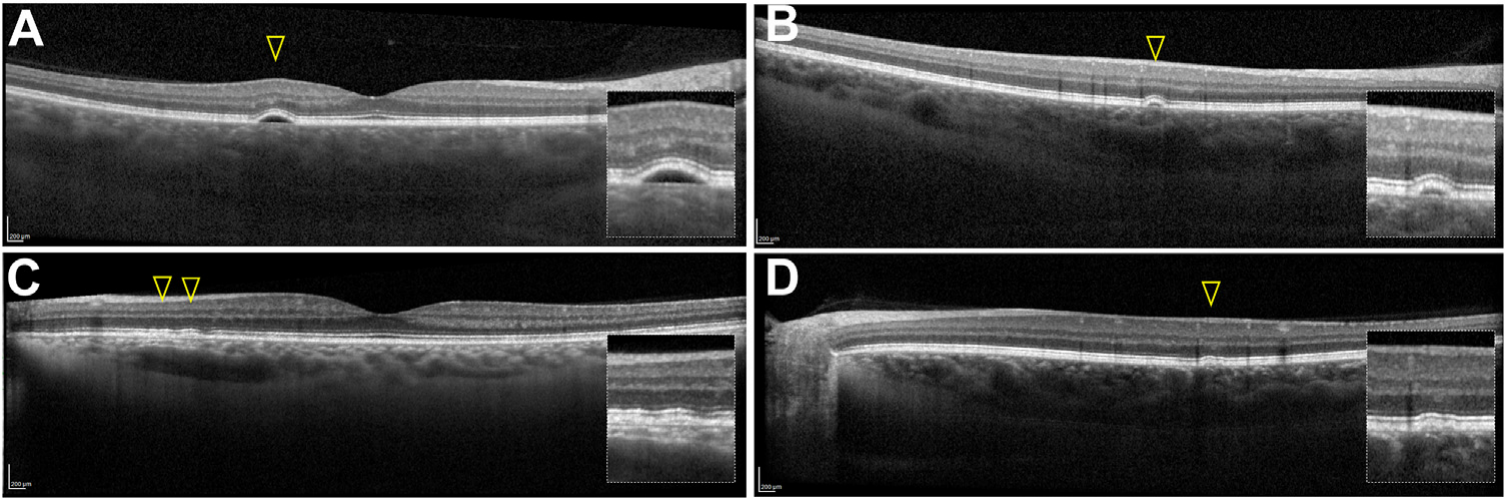
OCT features of PPE (arrowheads) overlying dilated choroidal vessels: **(A-B)** PEDs, **(C)** RPE elevations, and **(D)** RPE thickening. All findings were observed in the absence of pachychoroid-related exudation, such as subretinal fluid or subretinal hyperreflective material. PEDs = pigment epithelial detachments; PPE = pachychoroid pigment epitheliopathy; RPE =retinal pigment epithelium.

In our study, we report a similar but slightly lower prevalence of PPE in KC eyes (10.5%, 95% CL = 4.9/19.1%), which could be attributed to the different sample size between the 2 studies^6^ (approximately 1 out of 6 vs. 1 out of 10 patients, with overlapping confidence intervals). This difference in sample size may explain why we identified a significant difference in SFCT values between KC eyes with and without PPE: we hypothesize that the higher SFCT in the PPE group may contribute to RPE damage due to inner choroidal ischemia. Retinal pigment epithelium alterations in our series were characterized by multifocal pigment epithelial detachment (Fig. 3), RPE elevations (Fig. 4), and RPE thickening (Fig. 5), overlying regions of choroidal thickening, and fundus autofluorescence alterations.

A relevant additional aspect of this study is represented by the analysis of the sclera stroma, also known as the *substantia propria*^18^; whereas previous studies investigated KC versus healthy controls,^16^^,^^22^^,^^23^ we measured the temporal, nasal, and mean scleral thicknesses at 6-mm eccentricity in PPE-positive versus PPE-negative eyes within the KC population (453 vs. 382 μm, *P* = 0.013). Interestingly, while scleral thickening is increasingly recognized as a relevant pathogenic factor contributing to the manifestations of chorioretinal diseases, including PDS disorders^9^^,^^10^ and inflammatory conditions,^24^ our findings suggest a specific association in PPE eyes accompanied by KC.

Despite their differing optical properties, the cornea and sclera share a common embryological origin in mesenchymal cells and show similar collagen compositions.^22^ Both connective structures contain a high prevalence of type I collagen, along with fibrils of types III, V, VI, and XII chains, which are present in both corneal and scleral tissues.^25^ Besides altered collagen expression and deficient interlacing, KC causes dysregulation of proteoglycans^26^ and glycosaminoglycans (GAGs)^27^ in the extracellular collagenous matrix. The extracellular collagenous matrix plays a pivotal role in the development of the corneoscleral envelope, including cornea, sclera, and lamina cribrosa, and is also a constituent of retinal and choroidal vessel walls.^28^^,^^29^ Specifically, an accumulation of GAG polyanions occurs in keratoconic corneas,^30^ accompanied by important changes affecting both core proteins and GAG chains.^31^ Given the similarities between corneal and scleral extracellular collagenous matrix,^32^ we speculate that the altered GAG composition may predispose to choroidal and scleral thickening observed in PPE patients through a reduced transscleral protein outflow.^33^

There are currently 2 theories explaining the influence of scleral tissue in PDS^34^: (1) direct compression and strangulation of the intrascleral tract of vortex veins by a thick and rigid sclera, generating a bottleneck effect at the exit site of congested vortex veins^35^; and (2) a globally decreased transscleral macromolecular diffusion, leading to fluid loculations in the outer choroid^36^ and the suprachoroidal space.^37^ We are inclined to support this second mechanism, reported in cases of uveal effusion syndrome^33^^,^^38^ and corroborated by the evidence that the human sclera exhibits maximum thickness at the posterior pole.^39^ Reduced scleral hydraulic conductivity has been linked in uveal effusion syndrome to an increased concentration of GAGs, leading to a reduction of the normal transscleral egress of albumin with subsequent osmotic fluid retention.^38^

Our indocyanine green angiography findings across examined cases consistently demonstrated choroidal hyperpermeability with or without some mild degree of pathological or abnormal vortex vein dilation (see Fig 4), supporting our hypothesis that altered composition of the scleral tissues, most pronounced at the posterior pole where scleral domain thickness reaches its maximum, may drive the PPE development in a proportion of KC patients. Finally, the age-related component of this association suggests that biomechanical rather than purely anatomical factors may underlie the KC–PPE relationship. Our analysis revealed that patients without PPE were 10 years younger than those with PPE, suggesting better compensatory mechanisms of the RPE and choroidal vessels in the younger group, protecting against the PPE development, highlighting the active role of age-related healing and homeostasis.^40^

Our DT models revealed hierarchical feature importance in discriminating between PPE and non-PPE patients. The algorithm consistently identified increased scleral thickness as the dominant predictive feature (normalized Gini importance score: 0.646), followed by SFCT (0.286) and age (0.066). The confusion matrix showed high specificity (87.4% true negatives) but lower sensitivity (72.7% true positives) in PPE detection, a performance pattern reflecting the class imbalance in our dataset (Fig 6). These findings suggest that the combination of increased scleral thickness, choroidal thickness, and advancing age forms a significant risk profile for PPE development. The clinical relevance of our findings lies in their potential to refine screening protocols for KC patients based on these 3 salient features. Anterior scleral thickness measurement, readily obtainable during routine anterior segment OCT examination in horizontal gaze positions, may serve as an additional biomarker warranting more comprehensive posterior segment evaluation.

The limitations of this study must be acknowledged, primarily due to its retrospective cross-sectional design, the absence of a control group of age-matched individuals without KC, and the relatively low prevalence of PPE events in our cohort (11 out of 170 eyes). It should be noted that our findings are based on a Caucasian population and may not be generalizable to other ethnic groups. Despite the existence of several staging systems for KC based on Scheimpflug tomography and biomechanics, our study did not categorize patients into specific stages, as previous reports highlight limitations of these staging systems in clinical practice and limited clinical significance of KC severity in PPE development.^6^ Nevertheless, to provide readers with a general impression of our sample, we classified our PPE-positive cases using the Amsler–Krumeich classification, revealing that PPE occurred across the spectrum of KC severity (54.5% stage 1; 27.3% stage 2; 9.1% stage 3; and 9.1% stage 4). This distribution further emphasizes the importance of posterior segment screening in KC patients regardless of disease severity. To ensure consistent analysis and greater homogeneity, we included only patients with stable KC at least 6 months post-epi-off cross-linking and with corneal thickness exceeding 400 μm. While Romano et al (2012) demonstrated no significant retinal morphological changes following CXL treatment,^41^ our study design cannot definitively exclude a potential association between CXL and PPE development. Future longitudinal studies should specifically evaluate whether PPE worsens after CXL treatment, as the current cross-sectional design limits our ability to establish such temporal relationships. Manual measurements of scleral thickness using spectral-domain anterior segment OCT represent another study limitation, although this approach has demonstrated excellent reproducibility in both healthy and KC eyes in previous studies.^15^^,^^16^ Nevertheless, the study's strengths include the application of strict inclusion criteria, the inclusion of both eyes from each patient in the analysis using a generalized estimating equations approach, and multimodal imaging examples illustrating PPE manifestations in KC cases.

In conclusion, we confirm the relatively high prevalence of PPE in keratoconic eyes, occurring approximately in about 1 out of 10 eyes, and the key role of scleral thickening in its development. Using complementary analytical methods, we provide novel data on the importance of scleral thickening in PPE vs. non-PPE eyes, further supporting the rationale for multimodal imaging–based screening of posterior segment structures in patients with KC. Future longitudinal studies should be performed to investigate the potential risk of exudative conversion of PPE in KC eyes and should aim to identify potential additional risk factors associated with its development and progression.

## Declaration of Generative AI and AI-Assisted Technologies in the Writing Process

During the preparation of this manuscript, the authors used Claude 3.7 Sonnet (**Anthropic**) to help improve the readability and language of specific sections of the manuscript. The AI tool was not used for data analysis or scientific interpretation. After using this tool, the authors thoroughly reviewed and edited the content as needed and take full responsibility for the content of the published article.
